# Beta-agonist drugs modulate the proliferation and differentiation of skeletal muscle cells *in vitro*

**DOI:** 10.1016/j.bbrep.2021.101019

**Published:** 2021-05-18

**Authors:** Boimpoundi Eunice Flavie Ouali, Hao-Ven Wang

**Affiliations:** a Department of Life Sciences, College of Biosciences and Biotechnology, National Cheng Kung University, Tainan City, 701, Taiwan, ROC; b Center for Bioscience and Biotechnology, National Cheng Kung University, Tainan City, 701, Taiwan, ROC; c Marine Biology and Cetacean Research Center, National Cheng Kung University, Tainan City, 701, Taiwan, ROC

**Keywords:** Agonist drugs, C2C12, Proliferation, Differentiation, GM, Growth medium, DM, Differentiation Medium, MyHC, Myosin heavy chain, MyoD, Myoblast determination protein 1

## Abstract

Essentially employed for the treatment of airway obstructions in humans, β-agonists are also known to have an anabolic effect in animals’ skeletal muscle. *In vivo* and *in vitro* studies have attested the increase in animal body mass and the hypertrophy of muscle cells following the administration of specific β-agonists. However, the contribution of β-agonists to C2C12 myoblasts growth remains obscure. We therefore aimed to investigate the impact of β1-and β2-agonist drugs on the proliferation and differentiation of skeletal muscle cells. Direct observations and cytotoxicity assay showed that clenbuterol, salbutamol, cimaterol and ractopamine enhanced muscle cell growth and viability during the proliferation stage. Structural examinations coupled to Western blot analysis indicated that salbutamol and cimaterol triggered a decrease in myotube formation. A better comprehension of the effect of β-agonists on myogenic regulatory genes in the muscle cells is crucial to establish a specific role of β-agonists in muscle development, growth, and regeneration.

## Highlights

• Clenbuterol, salbutamol, cimaterol, and ractopamine induce molecular responses in C2C12.
• Beta-agonists promote cell's attachment, viability, and proliferation during muscle cell proliferation stage.
• Salbutamol and cimaterol inhibit the formation of myotubes during differentiation stage.

## 1 Introduction

Agonists are drugs that mimic catecholamines, selectively activate adrenoceptor subtypes, and have several important therapeutic applications. Beta-adrenergic agonists also called β-agonists bind to β-receptors and bear important functions in bronchial smooth muscle, liver, kidneys [1,2], and skeletal muscle [3]. Upon binding to β-adrenergic receptors, agonist drugs activate a cascade of events leading to muscle contraction via the accumulation of cAMP and the movement of calcium within the sarcoplasmic reticulum [4,5]. cAMP signaling is also associated with muscle cell hypertrophy and regeneration [6].

Beta-agonists have an important economic impact as they are involved in livestock production. Due to their so-called “repartitioning effects” [3], oral administration of β-agonist drugs to animals had enhanced their growth and altered their body composition [4,7]. The anabolic effects of β-agonists had been confirmed by several *in vivo* and *in vitro* studies [3,8,9]. Moreover, certain β-agonists have also been engaged in muscle repair and restoration of muscle function after injury [3] as well as in myopathies [8].

During myogenesis, skeletal muscle cells proliferate and differentiate from mononucleated myoblast cells to elongated, multinucleated structures called myocytes, myotubes, or myofibers. Skeletal muscle cell differentiation is an organized process regulated by gene networks that are spatiotemporally induced or repressed during cell fusion [10]. For instance, myoblast determination protein 1 (MyoD), myogenic factor 5 (Myf5), myogenin, and myogenic regulatory factor 4 (MRF4) referred to as myogenic regulatory factors collectively promote the maturation of myoblast cells [11,12] similarly to myosin heavy chain (MyHC) [13].

The changes in endogenous gene expression during C2C12 myogenic differentiation that result in myotube maturation and hypertrophy have been studied [14]. It is also well documented that β-agonists enhance the growth of muscle, decrease fat deposition, and improve feed efficiency of animals [[15], [16], [17]]. In the recent years, various studies on β-agonists have been focused on the determination of the drug residues in animal wastes and animal-derived foods [[18], [19], [20]], with the objective to inform and minimize the risk to human health and environment. However, documentation about the molecular mechanisms underlying animal muscle growth remains scarce. Therefore, we examine the pathway connecting skeletal muscle cell myogenesis to the increase of animal muscle mass by investigating the importance and implication of β-agonist drugs in the molecular regulation of *in vitro* myogenesis.

## 2 Materials and methods

### 2.1 Cell culture and drug treatments

Mouse C2C12 myoblast cells (ATCC® CRL-1772™) were obtained from ATCC (USA) and cultured in growth medium (GM) constituted of complete Dulbecco's Modified Eagle's Medium (DMEM) supplemented with 10% Fetal Bovine Serum (FBS, Gibco), and 1% Penicillin/Streptomycin (P/S, Gibco). The cells were maintained in the proliferation stage in GM for 48 h then differentiation was induced by replacing the GM with differentiation medium (DM) containing DMEM supplemented with 2% horse serum (Gibco), and 1% P/S. The cells were incubated at 37 °C in a 5% CO_2_ and humidified atmosphere with medium renewal every two days.

Clenbuterol, albuterol sulfate (salbutamol), cimaterol, and ractopamine hydrochloride were all purchased from Sigma Aldrich. Clenbuterol and salbutamol drugs powder were dissolved by using filtered water and pure Dimethyl sulfoxide (DMSO, Amresco) solvent was used to dissolve cimaterol and ractopamine to the concentration of 100 mM. Aliquots of 10^−3^ M for each drug were prepared and stored at −20 °C. Cells cultured in 90 mm dish were recovered and split at different density in various culture vessels and maintained in GM supplemented or not with agonist drugs diluted into the culture medium to the final concentration of 10^−6^ M. To induce differentiation, the GM was replaced with DM also containing agonist drugs.

### 2.2 cAMP activity assay

The increase of intracellular cAMP levels was determined using the cAMP Elisa Kit from Abnova. Briefly, 1 × 10^5^ cells/well was used to seed a 12-well plate. After 24 h of incubation at standard conditions, the cells were treated for 30 min by replacing GM with fresh GM containing clenbuterol, salbutamol, cimaterol, and ractopamine at the final concentration of 10^−6^ M and 10^−7^ M. Cell lysates were collected and assayed following the manufacturer's instructions.

### 2.3 Cell's viability and proliferation assay

The colorimetric MTT assay was used to assess the viability of the cells treated with β-agonist drugs. The method employed was modified from two groups of investigators [21,22]. A set of 96 well plates were seeded with 1 × 10^4^ cells/well and incubated from one to eight days. Every day, the cultured medium was aspired out from a plate and 150 μL of 3-(4,5-Dimethylthiazol-2-yl)-2,5-Diphenyltetrazolium Bromide (MTT reagent) diluted in fresh medium, to a final concentration of 0.5 mg/ml, was added to each well. The plates were incubated at standard conditions for 4 h then the medium was removed and 100 μL Dimethyl Sulfoxide (DMSO, J.T. Baker) was added to each well, followed by 10 min incubation at room temperature and optical density (OD) measurement at 492 nm.

To visualize the cells’ proliferation, C2C12 cells were plated at the density of 1 × 10^5^ cells/60 mm dish. Beta-agonist drugs at the final concentration of 10^−6^ M were added to the GM at seeding and the cells were incubated for 7 days with medium renewal every two days. Differentiation was induced two days after seeding by replacing the GM with DM, and the density of the cells was examined on photographs taken with Nikon eclipse TS 100 inverted microscope (Nikon, Japan).

### 2.4 Myotubes formation analysis

The 24-well plate containing pre-coated coverslip with 0.1% gelatin was seeded with 2.5 × 10^4^ cells/well. The cells were maintained in GM and DM supplemented with drugs until differentiation day 7. The cells were then rinsed twice with Phosphate-buffered saline (PBS, Protech) and fixed with 70% ethanol for 10 min. Hematoxylin Gill II stain (Surgipath) was immediately added to the wells and the plate was incubated for 3 min at room temperature. The cells were rinsed twice with deionized water and stained with Eosin stain (Surgipath) for 10 s followed by four times rinsing with deionized water. Coverslips were quick rinsed with increasing ethanol series of 70%, 95%, and 100% and mounted on microscope slides. Images of six different and random spots were captured per coverslip on Nikon eclipse TS 100 microscope. For calculation, the fusion index was evaluated as the ratio of the number of nuclei within myotubes containing two or more nuclei to the total number of nuclei for the six fields [23].

### 2.5 Protein extraction and western blotting

C2C12 cells were used to seed 60 mm dishes to a density of 1 × 10^5^ cells/dish. β-agonist drugs were added to GM and DM and the cells were treated at seeding. Total proteins were extracted at two days incubation in GM (D0), one day after differentiation induction (D1), four days (D4), and seven days (D7) of differentiation. On the ice, the cells were detached using a scrapper in a mixture consisting of 1x RIPA buffer (Cell signaling), protease inhibitor cocktail (Roche), phosphatase inhibitor cocktail II (Sigma-Aldrich), and phosphatase inhibitor cocktail III (Sigma-Aldrich). Protein lysates were then centrifuged at 4 °C for 20 min under 12 000 rpm, and supernatant collected and stored at −20 °C for the next steps. Samples of 20 μg of total protein were loaded on a 10% sodium dodecyl sulfate-polyacrylamide gel electrophoresis (SDS-PAGE) and transferred to Polyvinylidene fluoride (PVDF) blotting membrane (Amersham Hybond, GE Healthcare). The membranes were then blocked with 5% non-fat milk for 1 h and first antibodies of Heavy Chain Cardiac Myosin (Genetex, GTX20015, 1: 1000), alpha Actinin 2 (Genetex, GTX103219, 1:3000), Myogenin (5FD) (Novus, NB100-56510, 1:1500), MyoD1 (phosphor Ser200) (Genetex, GTX50144, 1:500), and GAPDH (Genetex, GTX100118, 1: 100 000) were applied for overnight at 4 °C. Secondary antibodies of Anti-Mouse IgG (whole molecule)- Peroxidase (Sigma-Aldrich, A9044) and Peroxidase-conjugated AffiniPure Goat anti-Rabbit IgG (H + L) (Jackson Immuno Research, 133 997) were used and blots were revealed using Immobilon Western Chemiluminescent HRP Substrate (Millipore). Three independent experiments were performed, and the ImageJ program was used for protein densitometry analysis.

### 2.6 Statistical analysis

All the experiments were performed at least three times with three biological replicates and results were presented as mean with the standard error of the mean (±SEM). A comparison of data sets from treated and untreated cells was performed using independent Student's t-test or two-way ANOVA on SPSS. Variations were considered significant at *p* < 0.05.

## 3 Results

### 3.1 Beta-agonists induce accumulation of intracellular cAMP and increase the viability of muscle cells during proliferation stage

**Fig. 1:**
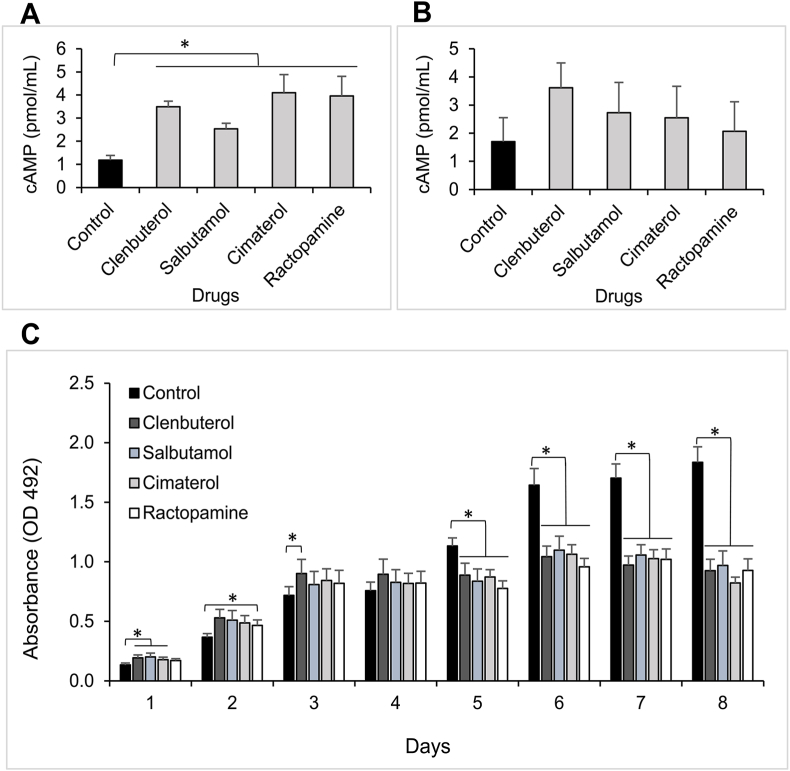
**Beta-agonists effect on cAMP production and proliferation of C2C12.** C2C12 cells treated with clenbuterol, salbutamol, cimaterol, and ractopamine at the final concentration of (A) 10^−6^ M and (B) 10^−7^ M for 30 min. Data are presented as mean ± SEM of three independent replicates and comparison between treated and untreated cells were assessed by using a Student's t-test. (C) MTT assay performed in triplicate at specific time points, from day 1 to day 8. Data are presented as mean ± SEM of four independent experiments with Two-way ANOVA analysis. (*) *p* < 0.05.

Cells treated with 10^−6^ M drugs showed a significant increase of cAMP concentration subsequent drug exposure (Fig. 1A) whereas drugs at 10^−7^ M had no substantial effects on the production of cAMP (Fig. 1B). For the next steps, we then considered 10^−6^ M agonist drugs as an adequate concentration for treating myoblast cells. By using the MTT method, viability of the cells was not impaired by the drugs. Fast proliferation with an increase of absorbance was noticed in treated cells compared to untreated cells at days 1, 2, and 3 (Fig. 1C). From day 5 to day 8, the trend was reversed with absorbance in control cells significantly greater than that of treated cells.

### 3.2 β-Agonists enhance cell attachment and proliferation

**Fig. 2:**
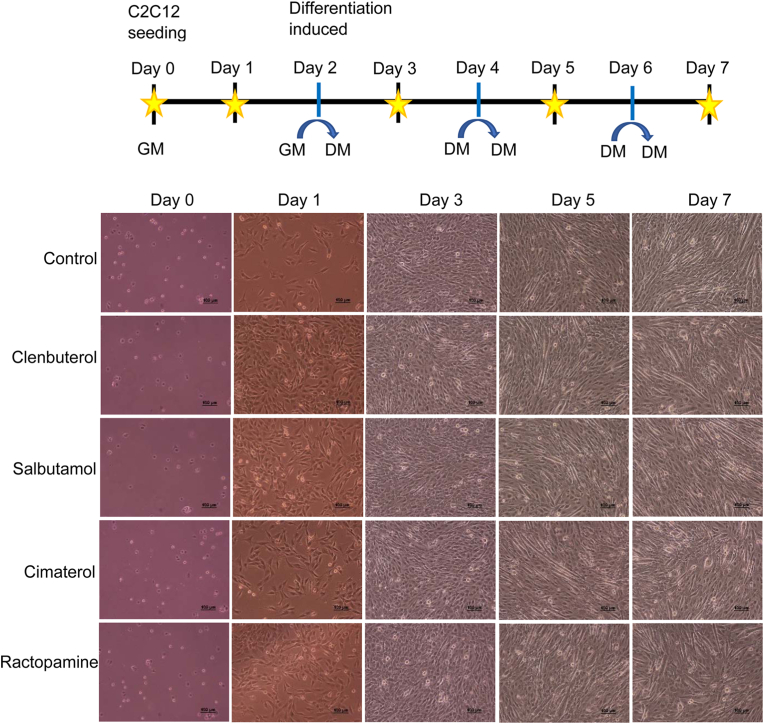
**C2C12 cells culture protocol and bright field observation.** Photographs of myoblast cells treated with 10^−6^ M clenbuterol, salbutamol, cimaterol, ractopamine and control cells were taken with Nikon eclipse TS 100 microscope at 10x magnification. Yellow stars show the specific time when the pictures were taken following the culture protocol. Scale bar: 100 μm. (For interpretation of the references to color in this figure legend, the reader is referred to the Web version of this article.)

All treated myoblast cells were able to form nascent myotubes at day 3 and mature myotubes at day 5 confirming the ability of the cells to grow and differentiate under agonist drugs (Fig. 2). Moreover, from an equal number of cells at seeding (day 0), cell density was greatly increased at day 1 in medium containing clenbuterol and salbutamol and moderately in medium containing ractopamine and cimaterol compared to the untreated (control) cells. These results suggested that β-agonist drugs may enhance attachment and proliferation of myoblast.

### 3.3 Salbutamol and cimaterol inhibit myotubes formation

**Fig. 3:**
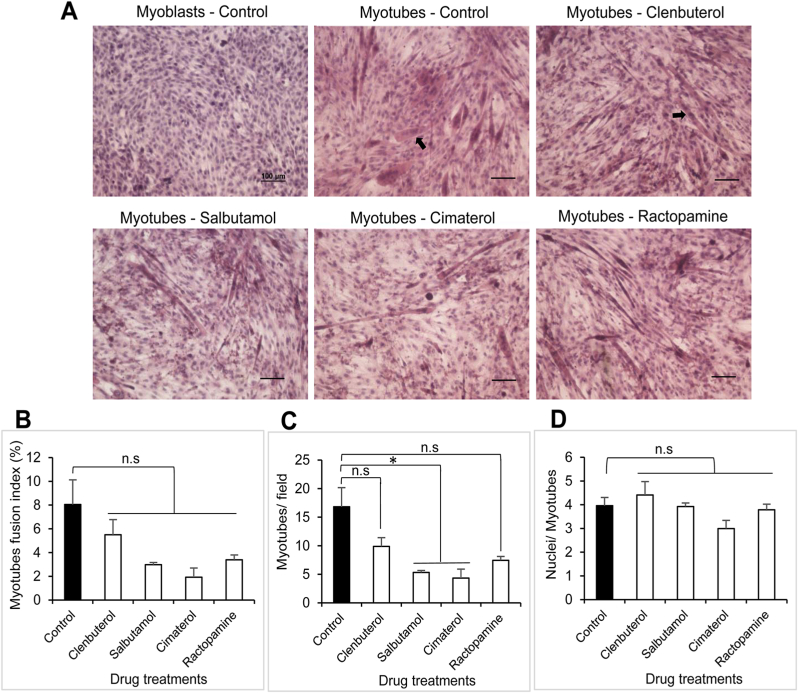
**Morphological changes induced by β-agonist drugs.** Hematoxylin and eosin staining of (A) myoblasts at differentiation day 0 and myotubes at differentiation day 7. Myotubes with darker pink cytoplasm contained multiple nuclei indicated in black arrows. Scale bar: 100 μm. (B) Myotubes fusion index represented the ratio of average nuclei inside myotubes to the total nuclei for six random fields/coverslip. (C) Average number of myotubes formed per field for each group of drug treatment. (D) Average number of nuclei within the myotubes. Data are presented as mean ± SEM of three independent experiments. Student's t-test was performed for statistical analysis. (*) *p* < 0.05, n. s: non-significant. (For interpretation of the references to color in this figure legend, the reader is referred to the Web version of this article.)

Myotubes formation was observed in the cells treated with β-agonist drugs as well as in untreated cells using H&E staining. The nucleus of myoblast cells at proliferation day 2 (differentiation day 0) were stained in blue color and the cytoplasm in light pink whereas multinucleated myotubes at differentiation day 7 had their cytoplasm colored in a darker pink (Fig. 3A). The fusion index of the cells treated with drugs decreased compared to that of control cells even if non-statistically supported (Fig. 3B). A significant decrease in the number of myotubes/field was noted with salbutamol- and cimaterol-treated cells (Fig. 3C). Yet, β-agonists had no effect in the average number of nuclei per myotubes (Fig. 3D).

### 3.4 The incidence of β-agonists on myogenic proteins expression during cell differentiation

**Fig. 4:**
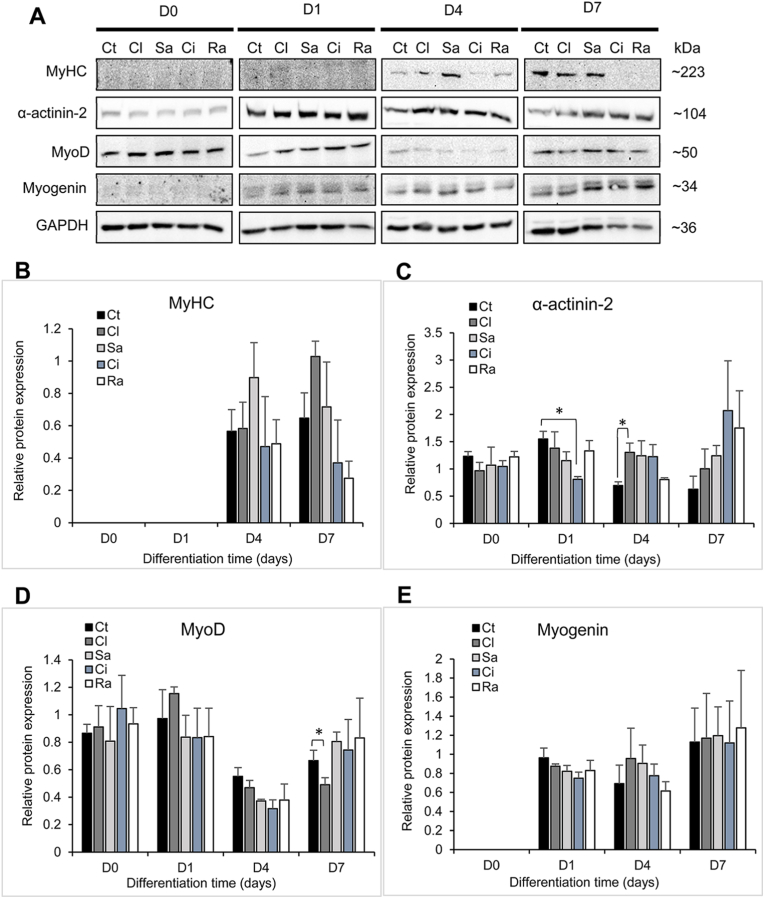
**Effect of β-agonists on myogenic proteins expression during cell differentiation.** (A) Western blot imaging of proteins extracted at differentiation days 0, 1, 4, and 7 from cells exposed to drugs at seeding. Densitometry analysis of (B) MyHC, (C) α-actinin-2, (D) MyoD, and (E) myogenin proteins normalized against GAPDH. Data are presented as mean ± SEM of three independent biological replicates. Ct: control, Cl: clenbuterol, Sa: salbutamol, Ci: cimaterol, Ra: ractopamine. (*) *p* < 0.05.

Total protein of cells treated with drugs at early (D0 and D1) and advanced (D4 and D7) differentiation stages were also analyzed on immunoblots to attest protein expression changes induced by β-agonists (Fig. 4A). All the cells expressed MyHC only at D4 and D7 but no statistical differences in the protein expression were noticed between treated and control cells (Fig. 4B). The expression level of α-actinin 2 was steady at D0 but considerably decreased at differentiation induction in cells exposed to cimaterol whereas clenbuterol-treated cells expressed an increased protein level in differentiating cells (Fig. 4C). A decreased level of MyoD in all treated cells was noticed at D4 even though non-statically supported whereas clenbuterol-treated cells presented a sustained decrease in the protein expression at D7 (Fig. 4D). Diversely, β-agonists did not significantly modulate the expression of myogenin during cell differentiation (Fig. 4E).

## 4 Discussion

Widely used for the treatment of airway disorders, β-agonists are also involved in animal meat production for human consumption. Herein, we focus on analyzing the effect of β-agonists on the proliferation and differentiation of muscle cell, a phenomenon that also occurs in a growing animal. Four drugs commonly used as agonists in animals and known to bind and activate the β1-and β2-adrenergic receptors of muscle cells were selected for this study. Clenbuterol and salbutamol are selective β2-adrenergic receptor agonists whereas cimaterol and ractopamine are nonselective agonists that activate both β1-and β2-adrenergic receptors [3,15,24]. Clenbuterol, salbutamol, and cimaterol, earlier used to promote broilers, pigs, sheep, and cattle meat production, are currently prohibited of use in animal farms [15,25,26]. On the other hand, the use of ractopamine is still approved for cattle in some countries [27].

From our results, the administration of 10^−6^ M β-agonist drugs to cultured cells significantly elevates the production of signaling molecule cAMP known to promote hypertrophic responses of muscle cells [6,28]. Similarly, various *in vitro* and *in vivo* studies have considered β-agonist drugs at the final concentration of 10^−6^ M as an optimal concentration inducing significant cellular responses in skeletal muscle cells [29,30]. Therefore, this concentration which also mimics drug doses orally administrated to animals *in vivo* [31,32] was maintained for downstream investigations. During proliferation stages, clenbuterol, salbutamol, cimaterol, and ractopamine were found to enhance attachment, growth, and viability of C2C12 cells. Simultaneously, the drugs exerted a cytotoxic activity when continuously administrated to the cultured cells during differentiation stages. In previous studies, mouse myoblast cell viability was found to decrease when treated with clenbuterol for 2 h [33] whereas 10^−7^ M salbutamol stimulated the proliferation of human airway epithelial cell lines in a time-dependent manner [34]. Very few studies have been conducted on the incidence of cimaterol and ractopamine on myoblastic cell line and to the best of our knowledge, no resources have discussed the action of these drugs on *Mus musculus* C2C12 viability. Direct microscope observations showed that not only β-agonists promote cell growth but also accelerate initial attachment of the cells to the culture vessels allowing cell proliferation and fusion into elongated myofiber. Furthermore, our results depicted the decrease of myotubes formation in cells treated with salbutamol and cimaterol during cell differentiation by comparison to the untreated cells. Myotubes formed in cimaterol-treated cells contained fewer cells than myotubes in control cells. These findings suggest that β-agonist drugs promote C2C12 proliferation but might inhibit the cell's differentiation.

Clenbuterol, salbutamol, cimaterol, and ractopamine are among the principal compounds in the group of β-agonists with anabolic effects in animals [1,15,35]. By supposing that β-agonists inhibit cells’ differentiation, we examined the protein expression pattern of myogenic factors and expected a decrease in the expression of MyHC, α-actinin 2, and myogenin at advanced differentiation and a decrease of MyoD protein expression at the early stage of myogenic differentiation. The Western blot quantitative results depicted reduced expression of α-actinin 2 in cells treated with cimaterol but an accumulation of α-actinin 2 in clenbuterol-treated cells which also correlate with a decreased level of MyoD protein [36]. The lack of substantial effect of the drugs on the expression of MyHC and myogenin in treated cells was similar to the observations described by Wannenes et al. [30]. Several investigations have reported the effect of cimaterol on lamb muscle hypertrophy [15,37] however no direct correlation of the β-agonist with skeletal cell line growth has indicated significant drug impact on myotube formation or cell fusion. In our case, it is possible that β-agonists have inhibitory or no effect on differentiation markers protein expression. The expression of various markers of skeletal muscle cells such as myogenin, MyoD, Myf5, Murf1, and MyHC were also analyzed by using the qPCR technique. The results showed that all the genes were differently expressed in treated and untreated cells with however extremely low levels of MyHC mRNA at differentiation day 5 in both control and treated cells (Appendix A, Appendix A). Variability in the *in vivo* and *in vitro* responses might be explained by the embryonic nature of the cells, the absence of vascular system and regulation of nutrient uptake, the lack of innervation, the inappropriate exposure to serum spectrum, and the possible indirect action of drugs that may ultimately model muscle hypertrophy [38]. The hypothesis that hormones in animals could also work in synergy with β-agonists to enhance muscle hypertrophy is not excluded and needs to be evaluated.

Our work emphasized that clenbuterol, salbutamol, cimaterol, and ractopamine promote C2C12 proliferation while inhibiting the viability of cells during the differentiation stage. Additionally, cimaterol inhibits myotubes formation. Thereby, the sustained β-agonists effect on animal cells may not rely on direct myogenic protein stimulation but either on indirect molecules interaction, hormones, or metabolic pathway triggered by the drugs. *In vitro* investigations, together with *in vivo* studies must be considered in their entirety to better comprehend the role of β-agonists on C2C12 myoblast cell differentiation.

## Funding information

This research did not receive any specific grant from funding agencies in the public, commercial, or not-for-profit sectors.

## Declaration of competing interest

The authors declare that they have no known competing financial interests or personal relationships that could have appeared to influence the work reported in this paper.

## Footnotes

Appendix A Supplementary data to this article can be found online at https://doi.org/10.1016/j.bbrep.2021.101019.
