# Supplementary material for: Beta-agonist drugs modulate the proliferation and differentiation of skeletal muscle cells in vitro
Source: Biochem Biophys Rep. 2021 May 18;26:101019. doi: 10.1016/j.bbrep.2021.101019 (PMC8144337; doi:10.1016/j.bbrep.2021.101019)
Supplement: Multimedia component 1 [file mmc1.pdf]

Supplementary data

Beta-agonist drugs modulate the proliferation and differentiation of skeletal muscle cells *in vitro*

Boimpoundi Eunice Flavie Ouali [a*], Hao-Ven Wang [a,b,c*]

[a] Department of Life Sciences, College of Biosciences and Biotechnology, National Cheng Kung University, Tainan City 701, Taiwan (R.O.C)

[b] Center for Bioscience and Biotechnology, National Cheng Kung University, Tainan City 701, Taiwan (R.O.C)

[c] Marine Biology and Cetacean Research Center, National Cheng Kung University, Tainan City 701, Taiwan (R.O.C)

[*]Co-corresponding authors:

Dr. Hao-Ven Wang

E-mail: hvwang@mail.ncku.edu.tw

National Cheng Kung University, Department of Life Sciences

No.1 University Road, East Dist., Tainan City 701, Taiwan (R.O.C)

Fax: (886) 6 2742538

Tel: (886) 6 2757575 ext. 58130

Dr. Boimpoundi Eunice Flavie Ouali

E-mail : l58047022@ncku.edu.tw

National Cheng Kung University, Department of Life Sciences

No.1 University Road, East Dist., Tainan City 701, Taiwan (R.O.C)

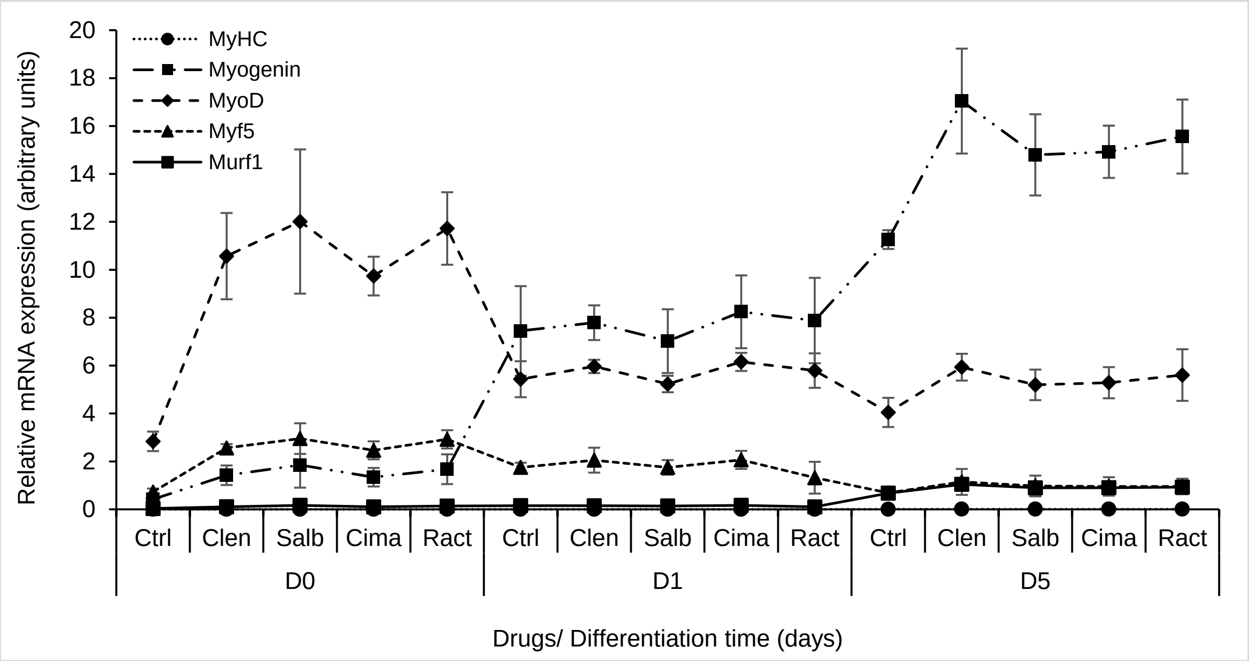

Fig. S1. Expression of myogenic regulatory factors in C2C12 cells following β-agonist treatments. qPCR was performed subsequent to cDNA synthesis and comparative CT (ΔΔCT) method was used for quantification of MyHC, myogenin, MyoD, Myf5 and Murf1 mRNA. Values were normalized against Ap3d1 CT values and data presented as mean ±SEM of three independent replicates. Ctrl: Control, Clen: clenbuterol, Salb: salbutamol, Cima: cimaterol, Ract: ractopamine.
